# 5,11-Dimethyl­dibenzo[*b*,*f*][1,5]diazocine-6,12(5*H*,11*H*)-dione

**DOI:** 10.1107/S1600536808001281

**Published:** 2008-01-18

**Authors:** Andrew B. Mahon, Paul Jensen, Andrew C. Try

**Affiliations:** a Department of Chemistry and Biomolecular Sciences, Building F7B, Macquarie University, NSW 2109, Australia; b Crystal Structure Analysis Facility, School of Chemistry, F11, University of Sydney, NSW 2006, Australia

## Abstract

In the mol­ecule of the title compound, C_16_H_14_N_2_O_2_, an *N*,*N*′-dimethyl­dianthranilide, the two methyl groups are disordered over two positions; site occupation factors were kept fixed as 0.75:0.25 and 0.65:0.35. The dihedral angle between the two benzene rings is 75.57 (3)°.

## Related literature

For related literature, see: Nadkarni & Hosangadi (1988 ▶). For related structures, see: Ebert *et al.* (1998 ▶); Nonnenmacher *et al.* (2000 ▶); Gordon-Wylie *et al.* (2004 ▶); Olszewska *et al.* (2004 ▶).
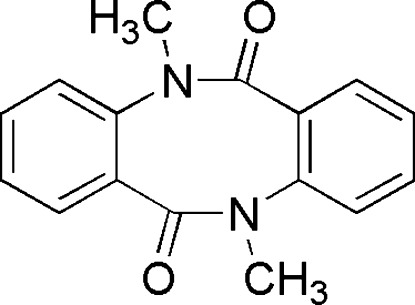


## Experimental

### 

#### Crystal data

C_16_H_14_N_2_O_2_
*M* *_r_* = 266.29
Monoclinic, 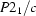

*a* = 11.2715 (10) Å
*b* = 7.9113 (7) Å
*c* = 15.4100 (14) Å
β = 101.611 (1)°
*V* = 1346.0 (2) Å^3^
*Z* = 4
Mo *K*α radiation
μ = 0.09 mm^−1^
*T* = 150 (2) K
0.55 × 0.42 × 0.24 mm

#### Data collection

Bruker SMART 1000 CCD area-detector diffractometer
Absorption correction: multi-scan (*SADABS*; Sheldrick, 1996 ▶) *T*_min_ = 0.901, *T*_max_ = 0.979
12897 measured reflections
3175 independent reflections
2686 reflections with *I* > 2σ(*I*)
*R*_int_ = 0.022

#### Refinement

*R*[*F* ^2^ > 2σ(*F* ^2^)] = 0.037
*wR*(*F* ^2^) = 0.104
*S* = 1.04
3175 reflections
185 parameters
1 restraint
H-atom parameters constrained
Δρ_max_ = 0.23 e Å^−3^
Δρ_min_ = −0.23 e Å^−3^

### 

Data collection: *SMART* (Bruker, 1998 ▶); cell refinement: *SAINT* (Bruker, 2003 ▶); data reduction: *SAINT*; program(s) used to solve structure: *SHELXS97* (Sheldrick, 2008 ▶); program(s) used to refine structure: *SHELXL97* (Sheldrick, 2008 ▶); molecular graphics: *X-SEED* (Barbour, 2001 ▶) and *SHELXTL* (Sheldrick, 2008 ▶); software used to prepare material for publication: *modiCIFer* (Guzei, 2005 ▶).

## Supplementary Material

Crystal structure: contains datablocks global, I. DOI: 10.1107/S1600536808001281/hk2415sup1.cif

Structure factors: contains datablocks I. DOI: 10.1107/S1600536808001281/hk2415Isup2.hkl

Additional supplementary materials:  crystallographic information; 3D view; checkCIF report

## Figures and Tables

**Fig. 1.**
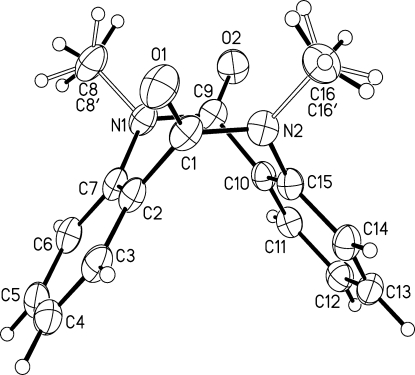
The molecular structure of the title molecule with the atom-numbering scheme. Displacement ellipsoids are drawn at the 50% probability level.

**Fig. 2.**
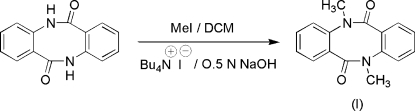
Synthetic scheme for the synthesis of (I).

## supplementary crystallographic information

### Comment

Several structures of the unsubstituted dianthranilide (*i.e.*, lacking methyl groups on the nitrogen atoms) are present in the literature, including ethanol, DMF and pyridine solvates of racemic material (Gordon-Wylie *et al.*, 2004) as well as a DMSO solvate of racemic material, unsolvated racemate and a DMSO solvate of enantiomerically pure crystals (Olszewska *et al.*, 2004).

In the molecule of the title compound, (I), (Fig. 1) the bond lengths and angles are within normal ranges. When the crystal structure was solved, the two methyl groups were found to be disordered. The dihedral angle between the two benzene rings is 75.57 (3)°.

The X-ray crystal structures of three *N*,*N*'-disubstituted dianthranilides have also been reported and in all cases they have a smaller dihedral angle between the two aryl rings of the dianthranilide in comparison with the structures of the unsubstituted compounds. The *N*,*N*'-di[1-(*N*-*t*-butylcarbamoyl)-1-(cyclohexyl)- methyl] (Ebert *et al.*, 1998), *N*,*N*'-dibenzyl (Nonnenmacher *et al.*, 2000) and *N*,*N*'-dicamphanoyl derivatives (Olszewska *et al.*, 2004) have dihedral angles of 78.2, 83.9 and 77.5°, respectively.

### Experimental

The title compound was prepared according to the literature procedure (Nadkarni & Hosangadi, 1988) in 89% yield. Single crystals of (I) were produced from slow evaporation of a dichloromethane solution.

### Refinement

When the crystal structure was solved, the two methyl groups were found to be disordered. They were each modelled with disorder over two positions with a common carbon atom. One was assigned a 75:25 split occupancy, the other 65:35. A rotating refinement was used for each methyl position giving staggered orientations for each. H atoms were positioned geometrically, with C—H = 0.95 and 0.98 Å for aromatic and methyl H atoms, respectively, and constrained to ride on their parent atoms, with *U*_iso_(H) = 1.2*U*_eq_(C).

### Crystal data

**Table:**

C_16_H_14_N_2_O_2_	*F*_000_ = 560
*M**_r_* = 266.29	*D*_x_ = 1.314 Mg m^−^^3^
Monoclinic, *P*2_1_/*c*	Melting point: 484 K
Hall symbol: -P 2ybc	Mo *K*α radiation λ = 0.71073 Å
*a* = 11.2715 (10) Å	Cell parameters from 5797 reflections
*b* = 7.9113 (7) Å	θ = 2.7–28.3º
*c* = 15.4100 (14) Å	µ = 0.09 mm^−^^1^
β = 101.611 (1)º	*T* = 150 (2) K
*V* = 1346.0 (2) Å^3^	Prism, colourless
*Z* = 4	0.55 × 0.42 × 0.24 mm

### Data collection

**Table:**

Bruker 1000 CCD area-detector diffractometer	3175 independent reflections
Radiation source: fine-focus sealed tube	2686 reflections with *I* > 2σ(*I*)
Monochromator: graphite	*R*_int_ = 0.022
*T* = 150(2) K	θ_max_ = 28.3º
ω scans	θ_min_ = 1.8º
Absorption correction: multi-scan(SADABS; Sheldrick, 1996)	*h* = −14→14
*T*_min_ = 0.901, *T*_max_ = 0.979	*k* = −10→10
12897 measured reflections	*l* = −20→20

### Refinement

**Table:**

Refinement on *F*^2^	Secondary atom site location: difference Fourier map
Least-squares matrix: full	Hydrogen site location: inferred from neighbouring sites
*R*[*F*^2^ > 2σ(*F*^2^)] = 0.037	H-atom parameters constrained
*wR*(*F*^2^) = 0.104	*w* = 1/[σ^2^(*F*_o_^2^) + (0.0528*P*)^2^ + 0.3606*P*] where *P* = (*F*_o_^2^ + 2*F*_c_^2^)/3
*S* = 1.04	(Δ/σ)_max_ < 0.001
3175 reflections	Δρ_max_ = 0.23 e Å^−^^3^
185 parameters	Δρ_min_ = −0.23 e Å^−^^3^
1 restraint	Extinction correction: none
Primary atom site location: structure-invariant direct methods	

### Special details

**Table:**

Geometry. All e.s.d.'s (except the e.s.d. in the dihedral angle between two l.s. planes) are estimated using the full covariance matrix. The cell e.s.d.'s are taken into account individually in the estimation of e.s.d.'s in distances, angles and torsion angles; correlations between e.s.d.'s in cell parameters are only used when they are defined by crystal symmetry. An approximate (isotropic) treatment of cell e.s.d.'s is used for estimating e.s.d.'s involving l.s. planes.
Refinement. Refinement of *F*^2^ against ALL reflections. The weighted *R*-factor *wR* and goodness of fit *S* are based on *F*^2^, conventional *R*-factors *R* are based on *F*, with *F* set to zero for negative *F*^2^. The threshold expression of *F*^2^ > σ(*F*^2^) is used only for calculating *R*-factors(gt) *etc*. and is not relevant to the choice of reflections for refinement. *R*-factors based on *F*^2^ are statistically about twice as large as those based on *F*, and *R*- factors based on ALL data will be even larger.

### Fractional atomic coordinates and isotropic or equivalent isotropic displacement parameters (Å^2^)

**Table:**

	*x*	*y*	*z*	*U*_iso_*/*U*_eq_	Occ. (<1)
O1	−0.03631 (7)	0.20770 (12)	0.36470 (6)	0.0392 (2)	
O2	0.32157 (9)	0.56190 (11)	0.51403 (6)	0.0384 (2)	
N1	0.26191 (9)	0.43083 (12)	0.38154 (6)	0.0293 (2)	
N2	0.11807 (8)	0.19261 (12)	0.48395 (6)	0.0280 (2)	
C1	0.07223 (10)	0.19025 (14)	0.39558 (8)	0.0283 (2)	
C2	0.15986 (10)	0.16141 (14)	0.33568 (7)	0.0270 (2)	
C3	0.14642 (11)	0.01939 (15)	0.28107 (7)	0.0313 (3)	
H3	0.0832	−0.0590	0.2833	0.038*	
C4	0.22439 (12)	−0.00839 (16)	0.22364 (8)	0.0346 (3)	
H4	0.2150	−0.1059	0.1869	0.042*	
C5	0.31624 (12)	0.10641 (17)	0.21977 (7)	0.0356 (3)	
H5	0.3706	0.0864	0.1811	0.043*	
C6	0.32876 (11)	0.25014 (15)	0.27217 (8)	0.0323 (3)	
H6	0.3905	0.3299	0.2683	0.039*	
C7	0.25127 (10)	0.27809 (14)	0.33037 (7)	0.0271 (2)	
C8	0.23206 (13)	0.59028 (16)	0.33401 (9)	0.0410 (3)	0.75
H8A	0.2291	0.6813	0.3766	0.049*	0.75
H8B	0.1530	0.5801	0.2939	0.049*	0.75
H8C	0.2941	0.6162	0.2996	0.049*	0.75
C8'	0.23206 (13)	0.59028 (16)	0.33401 (9)	0.0410 (3)	0.25
H8D	0.2974	0.6720	0.3534	0.049*	0.25
H8E	0.1562	0.6349	0.3466	0.049*	0.25
H8F	0.2226	0.5707	0.2702	0.049*	0.25
C9	0.30500 (10)	0.43187 (14)	0.47035 (7)	0.0271 (2)	
C10	0.33394 (10)	0.26308 (13)	0.51321 (7)	0.0239 (2)	
C11	0.45333 (10)	0.22278 (15)	0.55095 (7)	0.0272 (2)	
H11	0.5166	0.2994	0.5460	0.033*	
C12	0.48022 (10)	0.07159 (15)	0.59559 (7)	0.0304 (3)	
H12	0.5619	0.0443	0.6210	0.036*	
C13	0.38820 (11)	−0.04021 (15)	0.60338 (7)	0.0309 (3)	
H13	0.4071	−0.1439	0.6342	0.037*	
C14	0.26895 (11)	−0.00143 (14)	0.56646 (7)	0.0291 (2)	
H14	0.2059	−0.0777	0.5723	0.035*	
C15	0.24198 (9)	0.14978 (14)	0.52073 (7)	0.0243 (2)	
C16	0.03856 (11)	0.22890 (17)	0.54596 (9)	0.0363 (3)	0.65
H16A	−0.0385	0.2743	0.5134	0.044*	0.65
H16B	0.0774	0.3121	0.5897	0.044*	0.65
H16C	0.0236	0.1246	0.5763	0.044*	0.65
C16'	0.03856 (11)	0.22890 (17)	0.54596 (9)	0.0363 (3)	0.35
H16D	−0.0348	0.1596	0.5309	0.044*	0.35
H16E	0.0163	0.3488	0.5421	0.044*	0.35
H16F	0.0810	0.2026	0.6064	0.044*	0.35

### Atomic displacement parameters (Å^2^)

**Table:**

	*U*^11^	*U*^22^	*U*^33^	*U*^12^	*U*^13^	*U*^23^
O1	0.0249 (4)	0.0477 (5)	0.0415 (5)	−0.0008 (4)	−0.0015 (3)	0.0076 (4)
O2	0.0516 (6)	0.0251 (4)	0.0385 (5)	−0.0088 (4)	0.0088 (4)	−0.0029 (3)
N1	0.0320 (5)	0.0229 (5)	0.0309 (5)	−0.0034 (4)	0.0012 (4)	0.0057 (4)
N2	0.0235 (4)	0.0306 (5)	0.0297 (5)	−0.0045 (4)	0.0045 (4)	0.0013 (4)
C1	0.0257 (5)	0.0237 (5)	0.0330 (6)	−0.0039 (4)	0.0004 (4)	0.0043 (4)
C2	0.0271 (5)	0.0272 (5)	0.0235 (5)	−0.0012 (4)	−0.0029 (4)	0.0057 (4)
C3	0.0334 (6)	0.0278 (6)	0.0283 (5)	−0.0026 (4)	−0.0045 (4)	0.0047 (4)
C4	0.0440 (7)	0.0319 (6)	0.0242 (5)	0.0037 (5)	−0.0023 (5)	0.0014 (5)
C5	0.0417 (7)	0.0416 (7)	0.0232 (5)	0.0062 (5)	0.0055 (5)	0.0074 (5)
C6	0.0341 (6)	0.0349 (6)	0.0267 (5)	−0.0022 (5)	0.0036 (4)	0.0108 (5)
C7	0.0302 (5)	0.0257 (5)	0.0226 (5)	−0.0011 (4)	−0.0014 (4)	0.0064 (4)
C8	0.0490 (8)	0.0270 (6)	0.0428 (7)	−0.0010 (5)	−0.0012 (6)	0.0110 (5)
C8'	0.0490 (8)	0.0270 (6)	0.0428 (7)	−0.0010 (5)	−0.0012 (6)	0.0110 (5)
C9	0.0255 (5)	0.0248 (5)	0.0311 (6)	−0.0062 (4)	0.0055 (4)	0.0019 (4)
C10	0.0268 (5)	0.0245 (5)	0.0204 (5)	−0.0039 (4)	0.0044 (4)	−0.0015 (4)
C11	0.0253 (5)	0.0316 (6)	0.0245 (5)	−0.0051 (4)	0.0047 (4)	−0.0022 (4)
C12	0.0277 (5)	0.0365 (6)	0.0257 (5)	0.0022 (4)	0.0024 (4)	−0.0006 (4)
C13	0.0392 (6)	0.0279 (6)	0.0247 (5)	0.0016 (5)	0.0045 (4)	0.0038 (4)
C14	0.0333 (6)	0.0277 (6)	0.0258 (5)	−0.0071 (4)	0.0046 (4)	0.0016 (4)
C15	0.0244 (5)	0.0262 (5)	0.0217 (5)	−0.0042 (4)	0.0031 (4)	−0.0008 (4)
C16	0.0297 (6)	0.0417 (7)	0.0390 (7)	−0.0057 (5)	0.0107 (5)	−0.0040 (5)
C16'	0.0297 (6)	0.0417 (7)	0.0390 (7)	−0.0057 (5)	0.0107 (5)	−0.0040 (5)

### Geometric parameters (Å, °)

**Table:**

O1—C1	1.2280 (13)	C6—H6	0.9500
O2—C9	1.2229 (14)	C8—H8A	0.9800
N1—C9	1.3569 (15)	C8—H8B	0.9800
N1—C7	1.4346 (15)	C8—H8C	0.9800
N1—C8	1.4635 (14)	C9—C10	1.4964 (15)
N2—C1	1.3558 (15)	C10—C11	1.3913 (15)
N2—C15	1.4380 (14)	C10—C15	1.3926 (14)
N2—C16	1.4642 (15)	C11—C12	1.3827 (16)
C1—C2	1.4990 (16)	C11—H11	0.9500
C2—C3	1.3936 (16)	C12—C13	1.3867 (17)
C2—C7	1.3980 (15)	C12—H12	0.9500
C3—C4	1.3846 (18)	C13—C14	1.3844 (17)
C3—H3	0.9500	C13—H13	0.9500
C4—C5	1.3874 (19)	C14—C15	1.3906 (15)
C4—H4	0.9500	C14—H14	0.9500
C5—C6	1.3854 (18)	C16—H16A	0.9800
C5—H5	0.9500	C16—H16B	0.9800
C6—C7	1.3897 (17)	C16—H16C	0.9800
			
C9—N1—C7	122.20 (9)	N1—C8—H8A	109.5
C9—N1—C8	119.94 (10)	N1—C8—H8B	109.5
C7—N1—C8	117.74 (9)	N1—C8—H8C	109.5
C1—N2—C15	122.43 (9)	O2—C9—N1	122.97 (10)
C1—N2—C16	119.90 (10)	O2—C9—C10	120.80 (10)
C15—N2—C16	117.55 (9)	N1—C9—C10	116.21 (9)
O1—C1—N2	122.43 (11)	C11—C10—C15	119.42 (10)
O1—C1—C2	120.47 (10)	C11—C10—C9	119.71 (9)
N2—C1—C2	117.09 (9)	C15—C10—C9	120.75 (10)
C3—C2—C7	119.27 (11)	C12—C11—C10	120.20 (10)
C3—C2—C1	119.31 (10)	C12—C11—H11	119.9
C7—C2—C1	121.35 (10)	C10—C11—H11	119.9
C4—C3—C2	120.54 (11)	C11—C12—C13	120.13 (10)
C4—C3—H3	119.7	C11—C12—H12	119.9
C2—C3—H3	119.7	C13—C12—H12	119.9
C3—C4—C5	119.91 (11)	C14—C13—C12	120.29 (11)
C3—C4—H4	120.0	C14—C13—H13	119.9
C5—C4—H4	120.0	C12—C13—H13	119.9
C6—C5—C4	120.11 (12)	C13—C14—C15	119.60 (10)
C6—C5—H5	119.9	C13—C14—H14	120.2
C4—C5—H5	119.9	C15—C14—H14	120.2
C5—C6—C7	120.21 (11)	C14—C15—C10	120.36 (10)
C5—C6—H6	119.9	C14—C15—N2	119.89 (9)
C7—C6—H6	119.9	C10—C15—N2	119.71 (10)
C6—C7—C2	119.94 (11)	N2—C16—H16A	109.5
C6—C7—N1	119.66 (10)	N2—C16—H16B	109.5
C2—C7—N1	120.34 (10)	N2—C16—H16C	109.5
			
C15—N2—C1—O1	171.43 (10)	C7—N1—C9—O2	175.40 (11)
C16—N2—C1—O1	−4.45 (17)	C8—N1—C9—O2	−0.55 (18)
C15—N2—C1—C2	−7.30 (15)	C7—N1—C9—C10	−3.60 (15)
C16—N2—C1—C2	176.82 (10)	C8—N1—C9—C10	−179.55 (10)
O1—C1—C2—C3	−60.48 (15)	O2—C9—C10—C11	−64.70 (15)
N2—C1—C2—C3	118.28 (11)	N1—C9—C10—C11	114.32 (11)
O1—C1—C2—C7	116.44 (12)	O2—C9—C10—C15	111.25 (12)
N2—C1—C2—C7	−64.81 (14)	N1—C9—C10—C15	−69.72 (14)
C7—C2—C3—C4	1.56 (16)	C15—C10—C11—C12	−0.08 (16)
C1—C2—C3—C4	178.54 (10)	C9—C10—C11—C12	175.93 (10)
C2—C3—C4—C5	−0.46 (17)	C10—C11—C12—C13	−0.36 (16)
C3—C4—C5—C6	−1.08 (17)	C11—C12—C13—C14	0.11 (17)
C4—C5—C6—C7	1.52 (17)	C12—C13—C14—C15	0.59 (17)
C5—C6—C7—C2	−0.41 (16)	C13—C14—C15—C10	−1.03 (16)
C5—C6—C7—N1	−177.39 (10)	C13—C14—C15—N2	−178.59 (10)
C3—C2—C7—C6	−1.12 (15)	C11—C10—C15—C14	0.78 (16)
C1—C2—C7—C6	−178.04 (10)	C9—C10—C15—C14	−175.18 (10)
C3—C2—C7—N1	175.84 (9)	C11—C10—C15—N2	178.34 (9)
C1—C2—C7—N1	−1.08 (15)	C9—C10—C15—N2	2.37 (15)
C9—N1—C7—C6	−109.40 (12)	C1—N2—C15—C14	−108.33 (12)
C8—N1—C7—C6	66.63 (14)	C16—N2—C15—C14	67.64 (14)
C9—N1—C7—C2	73.63 (14)	C1—N2—C15—C10	74.10 (14)
C8—N1—C7—C2	−110.33 (12)	C16—N2—C15—C10	−109.93 (12)
